# Role of Multidetector Computed Tomography (CT) Virtual Hysteroscopy in the Evaluation of Abnormal Uterine Bleeding in Reproductive Age

**DOI:** 10.1155/2019/8910374

**Published:** 2019-01-21

**Authors:** Hend S. Saleh, Nadia M. Madkour, Ahmed Mahmoud Abdou, Entesar R. Mahdy, Hala E. Sherif, Ekramy A. Mohamed, Safaa A. Ibrahim, Mohamed I. Amin

**Affiliations:** ^1^Obstetrics & Gynecology Departments, Faculty of Medicine, Zagazig University, Zagazig 44519, Egypt; ^2^Radiodiagnosis Departments, Faculty of Medicine, Zagazig University, Zagazig 44519, Egypt

## Abstract

**Background:**

Abnormal uterine bleeding (AUB) may be acute or chronic and is defined as bleeding from the uterine corpus that is abnormal in regularity, volume, frequency, or duration and occurs in the absence of pregnancy. It is a widespread complaint in the primary care units. The prevalence of abnormal bleeding is up to 30% among women of reproductive age.

**Objective:**

To assess the role of CT virtual hysteroscopy in the evaluation of the uterine cavity in cases with abnormal uterine bleeding in reproductive age.

**Methods:**

Cross sectional study was performed at Obstetrics and Gynecology Department and Radiology Department, Zagazig University hospitals, Egypt, on 124 women with abnormal uterine bleeding in reproductive age, and their uterine cavity was evaluated by both row multidetector computed tomography (MDCT) scanner and Office hysteroscopy.

**Results:**

Mean age of studied group was 28.54 ± 5.99 years, and virtual hysteroscopy showed sensitivity 91.1% and specificity 85.3% in detection of abnormalities within uterine cavity. It showed sensitivity 91.1% and specificity 85.3% in cases of endometrial polyps. It yielded 88.5 % sensitivity and 100 % specificity in cases with submucous fibroids, while it yielded only 57.9 % sensitivity and 82.9% specificity in cases of thick endometrium.

**Conclusion:**

Virtual CT hysteroscopy is a good negative test in cases of abnormal uterine bleeding but has some limitations that decrease its sensitivity.

## 1. Introduction

Abnormal uterine bleeding is a common complaint in the primary care units. The prevalence of abnormal bleeding is up to 30% among women of reproductive age,** Singh et al., 2013 ** [1]. It is experienced by 15-20% of women attending outpatient gynecology clinics,** Matteson et al., 2009** [2].

The International Federation of Gynecology and Obstetrics (FIGO) had adopted a new classification system for abnormal uterine bleeding (AUB) in 2011 [3]. This system includes 9 main categories based on pattern of bleeding and its cause. American College of Obstetricians and Gynecologists (ACOG) accepted and published this classification system in 2013 [4].

This classification system is called PALM-COEIN. PALM stands for the pathologies associated with uterine structural anomalies (Polyps, Adenomyosis, Leiomyoma, Malignancy and endometrial hyperplasia), and COEIN stands for the pathologies not associated with uterine structural anomalies (coagulopathy, ovulatory dysfunction, endometrial causes, iatrogenic, not yet classified) [5].

AUB has been investigated with detail clinical history and clinical evaluation. There are various tools for diagnosis of AUB which are transvaginal sonography (TVS), saline infusion sonography, and hysteroscopy and confirmed on histopathology report. TVS has many advantages; being non-invasive, low-cost and appropriate for detection of uterine pathology. Thus, it has been recommended as a primary diagnostic tool for assessment pathology of uterine cavity in patients with AUB [6].

Saline infusion sonohysterography (SIS) entails injection of saline in uterine cavity during ultrasonographic examination. It increases the diagnostic accuracy of ultrasonographic evaluation of uterine cavity. Lesions distorting endometrial contour, endometrial polyps, and submucous fibroid are better recognized with SIS. Mathew et al. compared SIS to TVS and concluded that it has better sensitivity and negative predictive value than TVS alone [7].

Hysteroscopy is considered the gold standard method of evaluation of uterine cavity and cervical canal. It can also be used for treatment of some intracavitary pathologies [8].

Multidetector computed tomography (MDCT) had developed a revolutionary improved spatial and temporal resolutions, an effect making the virtual hysteroscopy possible [9]. Two and three dimensional and virtual endoscopic images allow the assessment of the endocervical canal and the interior of the uterine cavity, plus the advantage to get further details about extra uterine pelvic structures at the same exam [10]. Despite the main cornerstone of applying the virtual hysterosalpingography in investigations for female infertility, it can be used at other clinical conditions [11].

## 2. Aim of the Study

The aim of this study was to evaluate the role of virtual MDCT hysteroscopy in evaluation of the uterine cavity in patients with abnormal uterine bleeding during the reproductive period.

## 3. Patients and Methods

This study was conducted in the Radiology, Obstetrics and Gynecology Departments, Faculty of medicine, Zagazig University, Egypt. It included 124 women with abnormal bleeding during reproductive period selected from patients in the Department of Obstetrics and Gynecology.

Women with abnormal uterine bleeding in reproductive age were included in the study. Women with heavy vaginal bleeding affecting the general condition, other pelvic pathology (adnexal mass), pregnancy complication, and hormonal therapy were excluded from the study.

All eligible patients were properly counseled and gave written informed consent (filled a written survey including demographic and clinical data) before entry into the study. The protocol of the exam and the informed consent form used in this study were approved by the Institutional Review Board (IRB) of Zagazig University.

They were submitted to the following:

### 3.1. Virtual CT Hysteroscopy

#### 3.1.1. Patient Preparation

Patients were instructed to ask to empty the urinary bladder before the exam and get dressed a light cotton gown. Any undergarments and pads were removed. Holding breath during the exam is mandatory preventing motion artifact.

#### 3.1.2. CT Technique

The patient was laid on the CT couch in the lithotomy position. We used Povidone-iodine solution to clean the perineum. Dilation of the vagina through disposable plastic speculum to visualize the external os is the next step. 10-F plastic cannula was inserted into the external os of the cervix. Scout view of the pelvis was done. Fifteen ml of a iodine based diluted contrast material (2 ml ioxitalamate; Telebrix Hystero diluted with 13 ml saline solution) is injected through an automatic injector (A rate of 0.2 mL/sec). The slow flow rate was applied to reduce the patient's discomfort subsequently motion artifact and to secure good distension of the uterus. After beginning of contrast injection by 45 seconds, the scan is accomplished.

We used 128-row multidetector CT scanner (Ingenuity core 128 ™; Philips Medical Systems, Nederland). The scan parameters were as follows: section thickness, 0.5 mm; detector collimation, 128 × 0.625 mm; reconstruction interval, 0.25 mm; 100-120 kV; and scan time: 4 seconds. The tube current was automatically modulated (range, 120–250 mAs) and was applied to exhibit dose radiation reduction according to the size of the patient along the z-axis. The mean effective dose was 3.94± 0.5 milli-sievert (mSv).

#### 3.1.3. Imaging Processing

After acquisition of the row volume data, it was transferred to a dedicated workstation (Extended Brilliance Workspace; Philips Medical Systems- PHILIPS IntelliSpace Portal) to finalize the postprocessing.

MPR (Multiplanar reformat) in sagittal and coronal planes and CPR (Curved planar reformat) images were reconstructed through soft-tissue window settings.

Volume rendering (VR) reconstruction algorithm provides 3D views of the whole female internal genitalia, perfect at delineation of polyps, stenosis, and mural irregularities.

Virtual endoscopy postprocessing algorithm, when added to the 3D VR images together, is a powerful diagnostic imaging tool, providing intraluminal details.

### 3.2. Office Hysteroscopy

The hysteroscope used in our study was of Karl Storz (Germany). It is a rigid continuous flow panoramic hysteroscopy 25 cm in length and 2.9 mm in diameter, with an outer sheath of 3.2 mm and a 30 degree fibro-optic lens. The light source used in this study was a metal halide automatic light source from Circon Acmi G71A/Germany with a 150 Watt lamp.

Vaginoscopic approach was used. Uterine distension by saline 0.9% was done using hysteromate 3700 at pressure 50 mmHg.

A panoramic view of the uterine cavity to exclude any uterine malformations was done once the uterine cavity was entered. Examination was done systematically, first the fundus, anterior, posterior, and lateral walls of the uterus ending by visualization of the uterotubal junctions.

If there is any detected intrauterine pathology, the shape, the size and the site of it were evaluated. Because of optical distortion, mental correction was essential to detect the actual size properly. The thickness, the color, and vascularity of the endometrium were observed and recorded.

At the end of the procedure, the hysteroscope was slowly withdrawn through the cervical canal which was visualized to detect any intracervical pathology and to observe the shuttering mechanism of the internal os.

The results of virtual CT hysteroscopy were compared to the results of hysteroscopic examination as the gold standard and were subjected to statistical analysis to calculate sensitivity, specificity, positive predictive value, negative predictive value, diagnostic accuracy, and the degree of agreement with the hysteroscopy.

## 4. Statistical Analysis

Data collected throughout history, basic clinical examination, laboratory investigations and outcome measures coded were entered and analyzed using Microsoft Excel software. Data were then imported into Statistical Package for the Social Sciences (SPSS version 20.0)** (Statistical Package for the Social Sciences)** software for analysis.

**Sensitivity: **Ability of the test to detect true positive cases.

**Specificity:** Ability of the test to detect true negative cases.

**Positive predictive value:** Probability of disease in a patient with positive test results.

**Negative predictive value:** Probability of absence of disease in a patient with negative test result.

**Accuracy:** The proportion of all test results both positive and negative that are correct.

## 5. Results

A total of 124 females presented with abnormal uterine bleeding at the child bearing age were enrolled at this study. The mean age of studied group was 28.54 ± 5.99, and mean BMI was 25.67 ± 2.99. Most patients were multipara, 87 women (70.2%).

**Figure 1:**
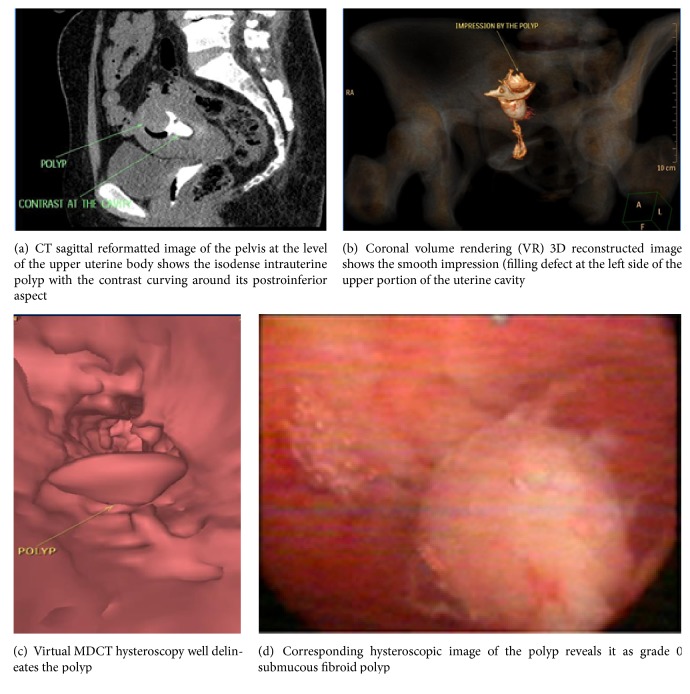


**Figure 2:**
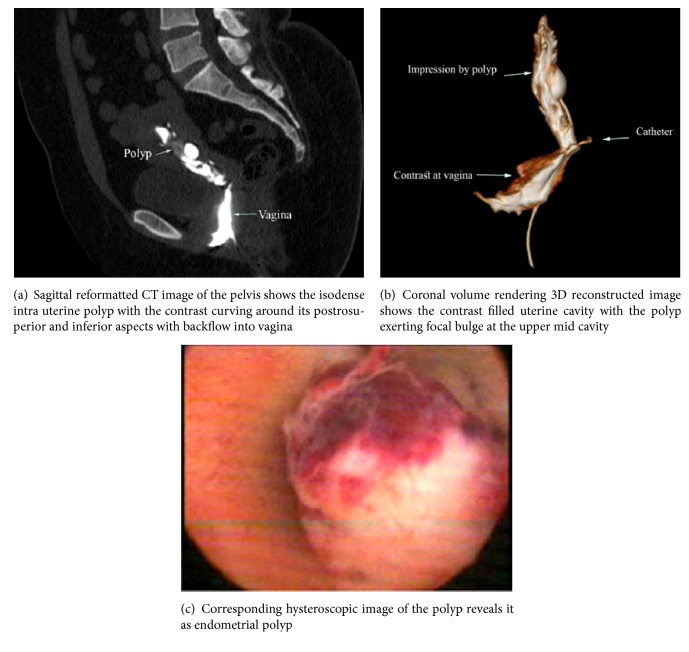


CT virtual hysteroscopy was in good agreement with hysteroscopy in detection of  (0.74) with sensitivity 91.1% and specificity 85.3% as shown in Table 1 and Figures 1(a), 1(b), 2(a), and 2(b).

Also it was in good agreement with hysteroscopy in detection of  (0.73) with sensitivity 84.4% and specificity 91.3% as shown in Table 2 and Figures 1(c), 1(d), and 2(c).

However, it was in very good agreement with hysteroscopy in detection of (0.92) with sensitivity 88.5% and specificity 100% as shown in Table 3.

It was in fair agreement with hysteroscopy in detection of  (0.33) with sensitivity 57.9% and specificity 82.9% as shown in Table 4.

No complication occurred during multidetector CT virtual hysteroscopy examination or office hysteroscopy.

30 % of the women at the reproductive age have been estimated to get an abnormal uterine bleeding (AUB). Those with AUB will suffer a load for their health, life style as well as the physicians dealing with such issue [9].

The advent of MDCT during the past years had made virtual CT studies rise to the surface of diagnostic imaging tools owing to the high-quality multiplanar, 3D reconstructions, and virtual endoscopic images, currently more feasible due to smaller slice thickness, together with high-spatial resolution. Our study was performed to depict the role of multidetector CT virtual hysteroscopy in the evaluation of abnormal uterine bleeding at the childbearing age. Not enough researches had been published at such topic till this moment. The imaging tools of abnormal uterine bleeding include transvaginal sonography (TVS), saline sonography, and hysteroscopy with confirmation by histopathology report in mass lesions. Hysteroscopy is a simple tool to explore the endocervical canal and uterine cavity. Additionally it could be used for treating multiple benign lesions. This study included thirty 124 female patients at the childbearing age, complaining of abnormal uterine bleeding. The mean age in our study was 28.54 ± 5.99 years, which was similar to** Ahmed et al 2014** [12] who performed a study with the mean age of 29.12 ± 5.5, although the target of their study was keen about infertility [5]. Meanwhile** Carrascosa et al., 2009, **carried out a study also about infertility with the mean age 35 years [10].

In this study, 23 of 26 cases (88.5%) with submucosal fibroid were correctly outlined by the CT virtual hysteroscopy with 88.5 % sensitivity and 100 % specificity when compared with hysteroscopy. These results were comparable with a study done by** Baronio et al., 2010,** that showed 97% and 100%, sensitivity and specificity, respectively [13].

On the basis of our results, we demonstrated that the MDCT virtual hysteroscopy has a good diagnostic performance in the assessment of the abnormal uterine bleeding, as it was in good agreement with hysteroscopy in detection of abnormalities within uterine cavity (0.74), sensitivity 91.1% and specificity 85.3%. It was in good agreement with hysteroscopy in detection of endometrial polyps (0.73), sensitivity 84.4% and specificity 91.3%.

However, it was in very good agreement with hysteroscopy in detection of submucous fibroids (0.92) with sensitivity 88.5% and specificity 100%, while it was in fair agreement with hysteroscopy in detection of thick endometrium (0.33) with sensitivity 57.9% and specificity 82.9%.

The capacity to evaluate the extra-uterine pelvic structures is an advantage of multidetector CT. It permits the detection of incidental findings, hence a big benefit to the patient [5]. One case at this study showed right side subserous fibroid that was not diagnosed at hysteroscopy but well delineated at CT exam.

Short procedure time is another advantage of the multidetector CT. According to our results, the exam time was 6 ± 0.9 min. This result is consistent with Carrascosa et al., 2010, who reported a mean duration of 5 ± 3 minutes. [11].

One of the drawbacks of the multidetector CT is the radiation exposure. Young patients (i.e., of reproductive age) have relative high risks from radiation exposure during the exam, especially with the focused CT beam on the gonadal region. To reduce the delivered radiation dose, automatic tube current modulation along the z-axis was applied. In this study, the mean effective radiation dose for virtual hysteroscopy performed through this method was 2.5 mSv.

## 6. Conclusion

Multidetector CT virtual hysteroscopy allowed a good detection of most of intra uterine pathologies responsible for abnormal uterine bleeding especially the submucous myomas. Limitation of the method includes relative weak sensitivity at delineation endometrial thickness, and additionally it carries a radiation risk compared to other radiation risk-free diagnostic tools.

## Figures and Tables

**Table 1:** Shows the diagnostic value of CT virtual hysteroscopy in evaluation of uterine cavity abnormalities.

			Hysteroscopy		total	Validity		X^2^	P	Kappa agreement
			Abnormal	Normal						
Virtual CT	Abnormal	N	**82**	**5**	**87**	Sensitivity	91.1%	68.8	0.00*∗∗*	0.74
		%	91.1%	14.7%	70.2%	Specificity	85.3%			
	Normal	N	**8**	**29**	**37**	PPV	94.2%			
		%	8.9%	85.3%	29.8%	NPV	78.3%			
Total		N	**90**	**34**	**124**	Accuracy	89.5%			
		%	100.0%	100.0%	100%					

**Table 2:** Shows the diagnostic value of CT virtual hysteroscopy in detection of endometrial polyps.

			Hysteroscopy		Total	Validity		X^2^	P	Kappa agreement
			Positive	Negative						
Virtual CT	Positive	N	**27**	**8**	**35**	Sensitivity	84.4%	67.12	0.00*∗∗*	0.73
		%	84.4%	8.7%	28.2%	Specificity	91.3%			
	Negative	N	**5**	**84**	**89**	PPV	77.1%			
		%	15.6%	91.3%	71.8%	NPV	94.3%			
Total		N	**32**	**92**	**124**	Accuracy	89.5%			
		%	100.0%	100.0%	100%					

**Table 3:** Shows the diagnostic value of CT virtual hysteroscopy in detection of submucous fibroid.

			Hysteroscopy		Total	Validity		X^2^	P	Kappa agreement
			Positive	Negative						
Virtual CT	Positive	N	**23**	**0**	**23**	Sensitivity	88.5%	106.4	0.00*∗∗*	0.92
		%	88.5%	0.0%	18.5%	Specificity	100.0%			
	Negative	N	**3**	**98**	**101**	PPV	100.0%			
		%	11.5%	100.0%	81.5%	NPV	97.02%			
Total		N	**26**	**98**	**124**	Accuracy	97.5%			
		%	100.0%	100.0%	100%					

**Table 4:** Shows the diagnostic value of CT virtual hysteroscopy in detection of thick endometrium.

			Hysteroscopy		Total	Validity		X^2^	P	Kappa agreement
			Positive	Negative						
Virtual CT	Positive	N	**11**	**18**	**29**	Sensitivity	57.9%	14.91	0.00*∗∗*	0.33
		%	57.9%	17.1%	23.4%	Specificity	82.9%			
	Negative	N	**8**	**87**	**95**	PPV	37.9%			
		%	42.1%	82.9%	76.6%	NPV	91.5%			
Total		N	**19**	**105**	**124**	Accuracy	79.03%			
		%	100.0%	100.0%	100%					

## Data Availability

The data used to support the findings of this study are available from the corresponding author upon request.
